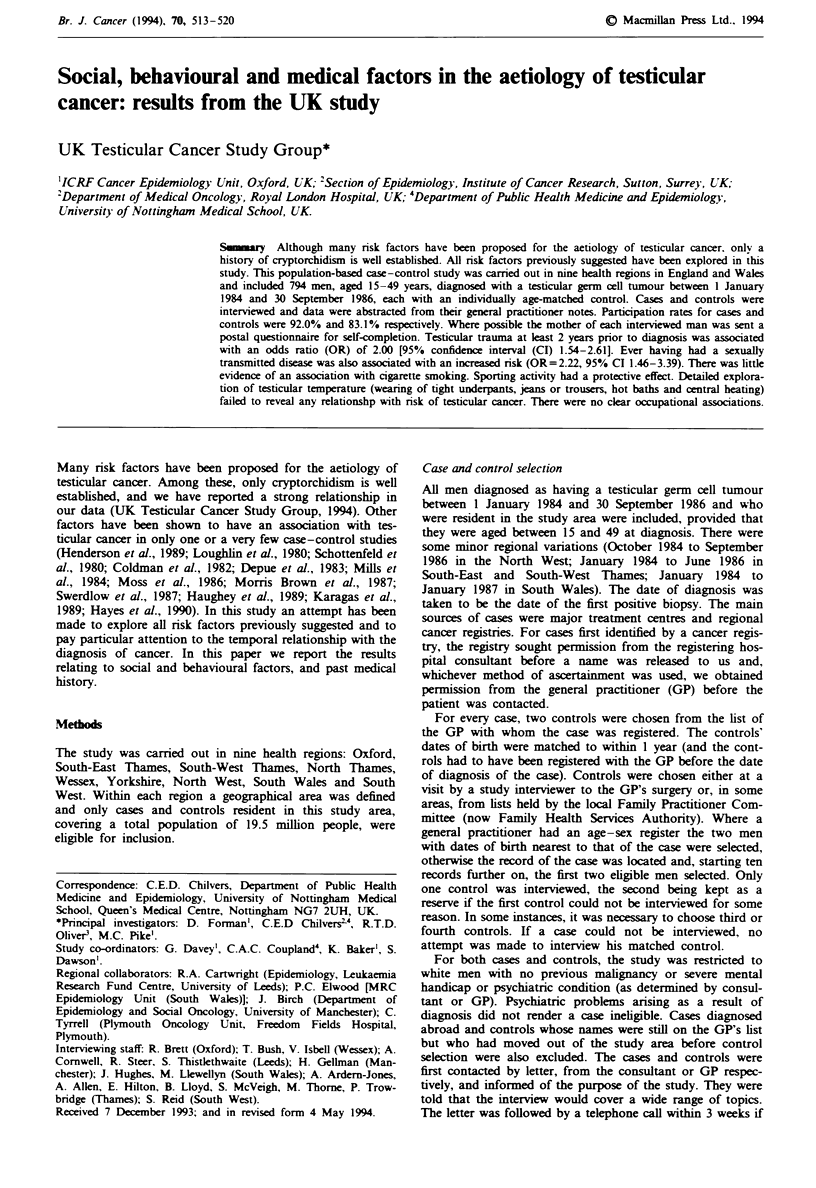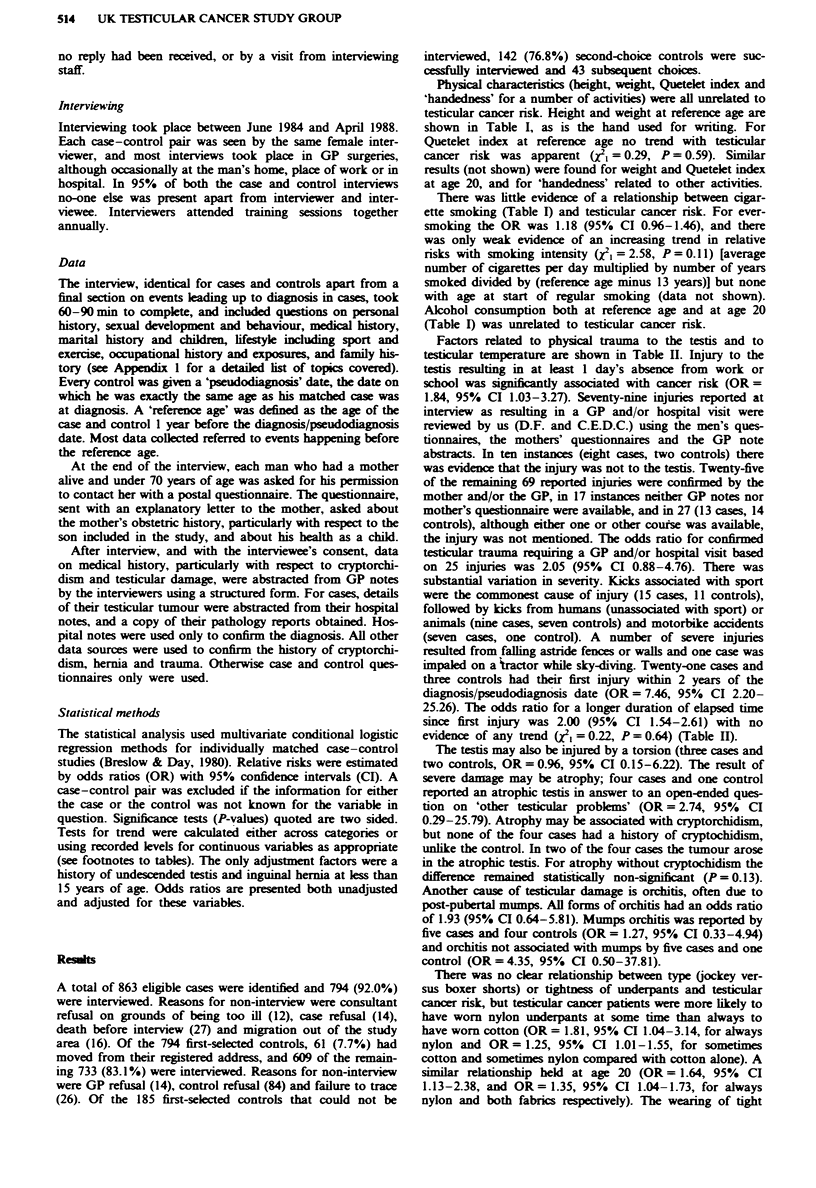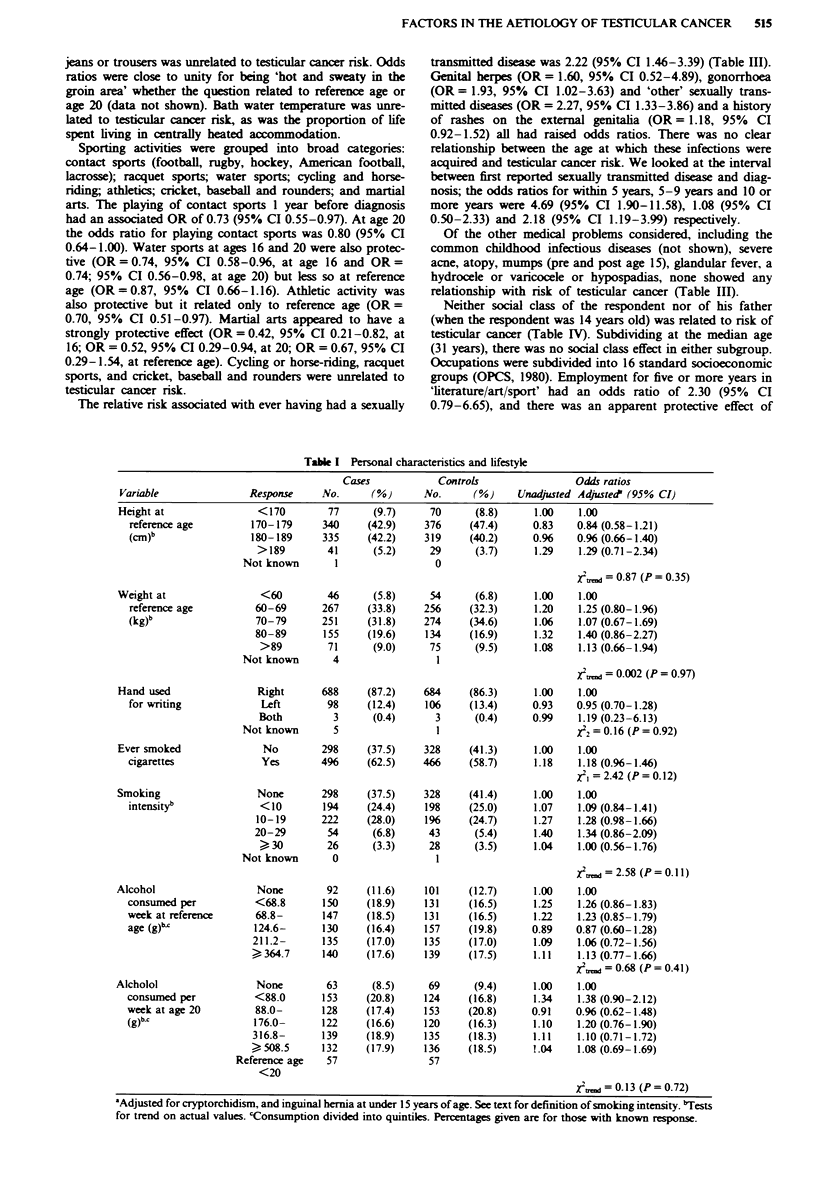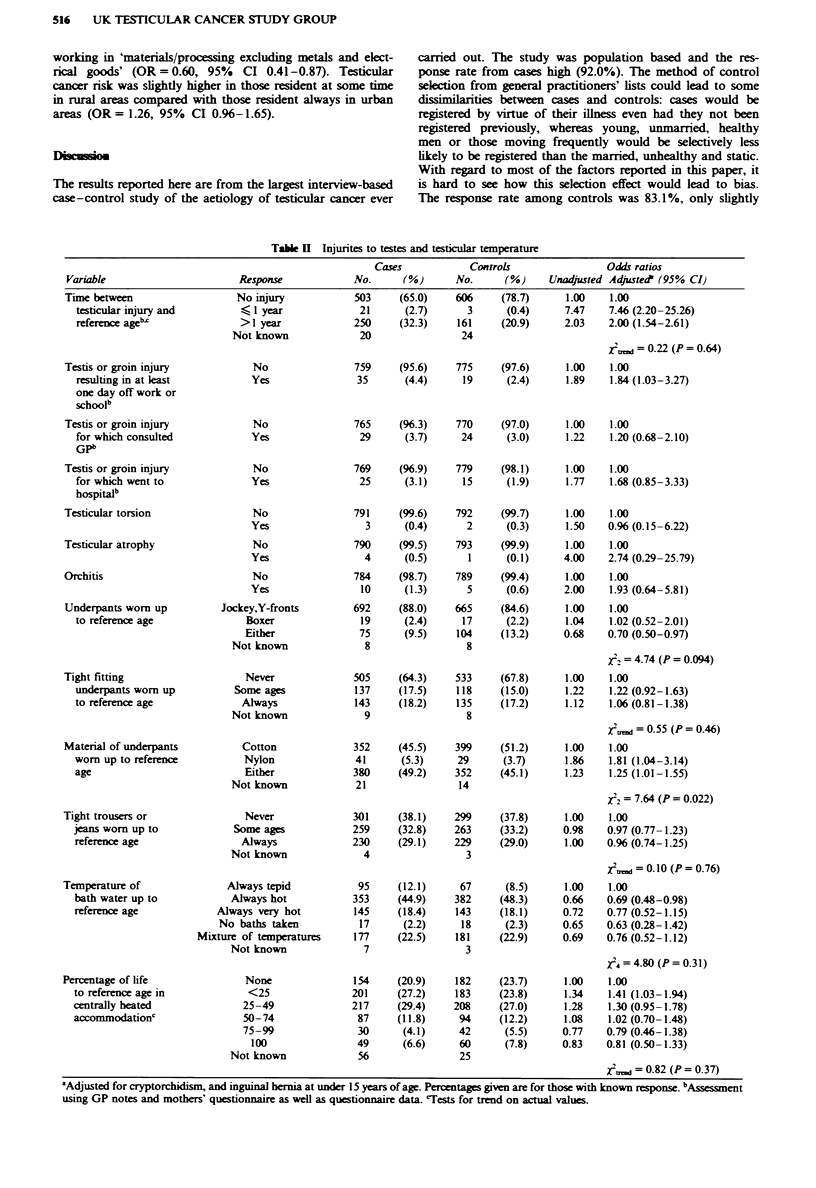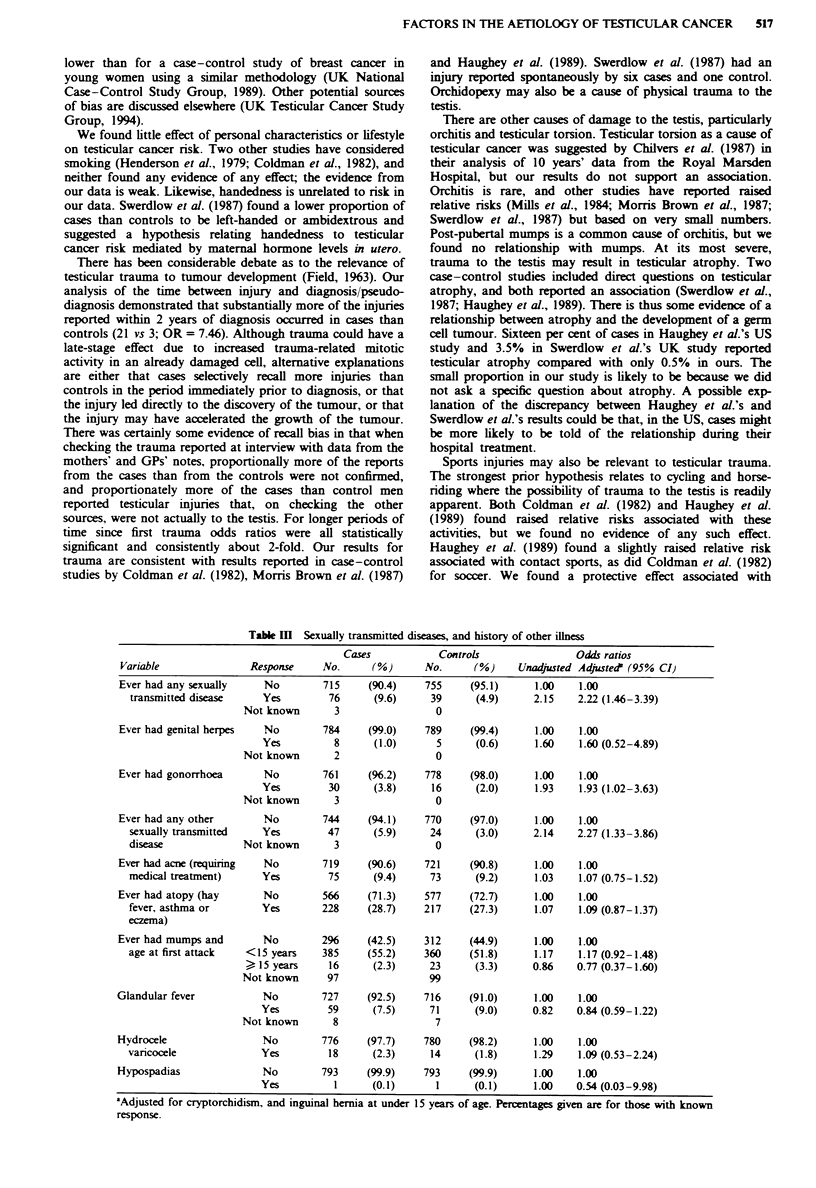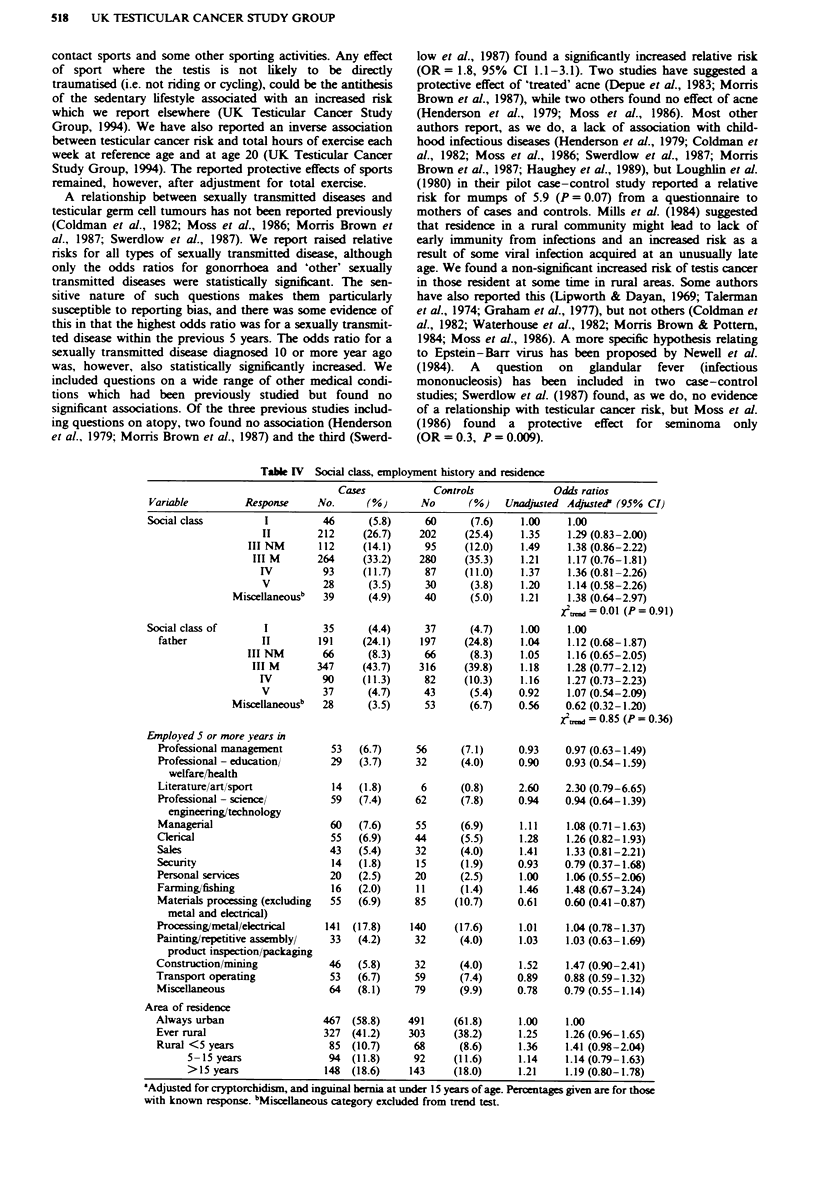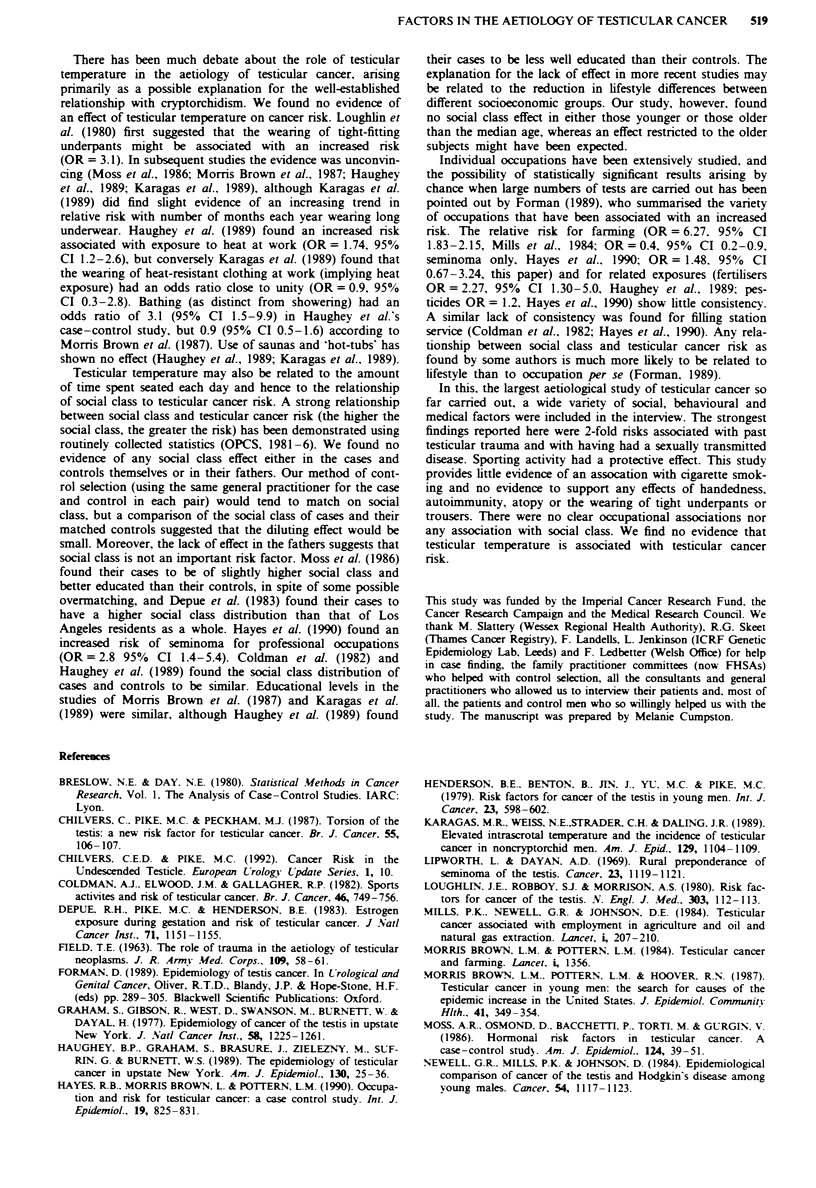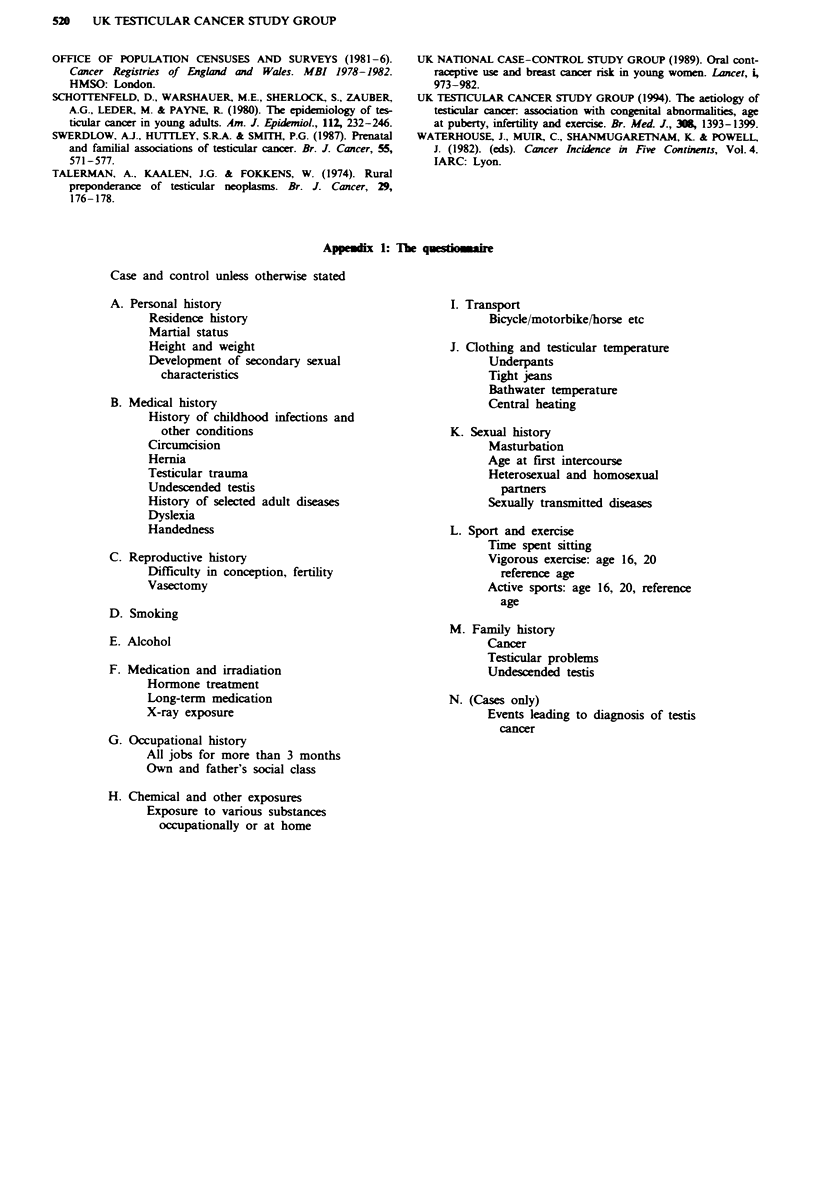# Social, behavioural and medical factors in the aetiology of testicular cancer: results from the UK study. UK Testicular Cancer Study Group.

**DOI:** 10.1038/bjc.1994.337

**Published:** 1994-09

**Authors:** 

## Abstract

Although many risk factors have been proposed for the aetiology of testicular cancer, only a history of cryptorchidism is well established. All risk factors previously suggested have been explored in this study. This population-based case-control study was carried out in nine health regions in England and Wales and included 794 men, aged 15-49 years, diagnosed with a testicular germ cell tumour between 1 January 1984 and 30 September 1986, each with an individually age-matched control. Cases and controls were interviewed and data were abstracted from their general practitioner notes. Participation rates for cases and controls were 92.0% and 83.1% respectively. Where possible the mother of each interviewed man was sent a postal questionnaire for self-completion. Testicular trauma at least 2 years prior to diagnosis was associated with an odds ratio (OR) of 2.00 [95% confidence interval (CI) 1.54-2.61]. Ever having had a sexually transmitted disease was also associated with an increased risk (OR = 2.22, 95% CI 1.46-3.39). There was little evidence of an association with cigarette smoking. Sporting activity had a protective effect. Detailed exploration of testicular temperature (wearing of tight underpants, jeans or trousers, hot baths and central heating) failed to reveal any relationship with risk of testicular cancer. There were no clear occupational associations.


Br. J. Cancer (1994), 70, 513-520 © Macmillan Press Ltd., 1994

### Social, behavioural and medical factors in the aetiology of testicular cancer: results from the UK study

UK Testicular Cancer Study Group*

[1]ICRF Cancer Epidemiology Unit, Oxford, UK; [2]Section of Epidemiology, Institute of Cancer Research, Sutton, Surrey, UK; [3]Department of Medical Oncology, Royal London Hospital, UK; [4]Department of Public Health Medicine and Epidemiology, University of Nottingham Medical School, UK.

**Summary** Although many risk factors have been proposed for the aetiology of testicular cancer, only a history of cryptorchidism is well established. All risk factors previously suggested have been explored in this study. This population-based case-control study was carried out in nine health regions in England and Wales and included 794 men, aged 15-49 years, diagnosed with a testicular germ cell tumour between 1 January 1984 and 30 September 1986, each with an individually age-matched control. Cases and controls were interviewed and data were abstracted from their general practitioner notes. Participation rates for cases and controls were 92.0% and 83.1% respectively. Where possible the mother of each interviewed man was sent a postal questionnaire for self-completion. Testicular trauma at least 2 years prior to diagnosis was associated with an odds ratio (OR) of 2.00 [95% confidence interval (CI) 1.54-2.61]. Ever having had a sexually transmitted disease was also associated with an increased risk (OR = 2.22, 95% CI 1.46-3.39). There was little evidence of an association with cigarette smoking. Sporting activity had a protective effect. Detailed exploration of testicular temperature (wearing of tight underpants, jeans or trousers, hot baths and central heating) failed to reveal any relationshp with risk of testicular cancer. There were no clear occupational associations.

Many risk factors have been proposed for the aetiology of testicular cancer. Among these, only cryptorchidism is well established, and we have reported a strong relationship in our data (UK Testicular Cancer Study Group, 1994). Other factors have been shown to have an association with testicular cancer in only one or a very few case-control studies (Henderson *et al.*, 1989; Loughlin *et al.*, 1980; Schottenfeld *et al.*, 1980; Coldman *et al.*, 1982; Depue *et al.*, 1983; Mills *et al.*, 1984; Moss *et al.*, 1986; Morris Brown *et al.*, 1987; Swerdlow *et al.*, 1987; Haughey *et al.*, 1989; Karagas *et al.*, 1989; Hayes *et al.*, 1990). In this study an attempt has been made to explore all risk factors previously suggested and to pay particular attention to the temporal relationship with the diagnosis of cancer. In this paper we report the results relating to social and behavioural factors, and past medical history.

### Methods

The study was carried out in nine health regions: Oxford, South-East Thames, South-West Thames, North Thames, Wessex, Yorkshire, North West, South Wales and South West. Within each region a geographical area was defined and only cases and controls resident in this study area, covering a total population of 19.5 million people, were eligible for inclusion.

Correspondence: C.E.D. Chilvers, Department of Public Health Medicine and Epidemiology, University of Nottingham Medical School, Queen's Medical Centre, Nottingham NG7 2UH, UK.
*Principal investigators: D. Forman[1], C.E.D Chilvers[2,4], R.T.D. Oliver[3], M.C. Pike[1].
Study co-ordinators: G. Davey[1], C.A.C. Coupland[4], K. Baker[1], S. Dawson[1].
Regional collaborators: R.A. Cartwright (Epidemiology, Leukaemia Research Fund Centre, University of Leeds); P.C. Elwood [MRC Epidemiology Unit (South Wales)]; J. Birch (Department of Epidemiology and Social Oncology, University of Manchester); C. Tyrrell (Plymouth Oncology Unit, Freedom Fields Hospital, Plymouth).
Interviewing staff: R. Brett (Oxford); T. Bush, V. Isbell (Wessex); A. Cornwell, R. Steer, S. Thistlethwaite (Leeds); H. Gellman (Manchester); J. Hughes, M. Llewellyn (South Wales); A. Ardern-Jones, A. Allen, E. Hilton, B. Lloyd, S. McVeigh, M. Thorne, P. Trowbridge (Thames); S. Reid (South West).
Received 7 December 1993; and in revised form 4 May 1994.

### *Case and control selection*

All men diagnosed as having a testicular germ cell tumour between 1 January 1984 and 30 September 1986 and who were resident in the study area were included, provided that they were aged between 15 and 49 at diagnosis. There were some minor regional variations (October 1984 to September 1986 in the North West; January 1984 to June 1986 in South-East and South-West Thames; January 1984 to January 1987 in South Wales). The date of diagnosis was taken to be the date of the first positive biopsy. The main sources of cases were major treatment centres and regional cancer registries. For cases first identified by a cancer registry, the registry sought permission from the registering hospital consultant before a name was released to us and, whichever method of ascertainment was used, we obtained permission from the general practitioner (GP) before the patient was contacted.

For every case, two controls were chosen from the list of the GP with whom the case was registered. The controls' dates of birth were matched to within 1 year (and the controls had to have been registered with the GP before the date of diagnosis of the case). Controls were chosen either at a visit by a study interviewer to the GP's surgery or, in some areas, from lists held by the local Family Practitioner Committee (now Family Health Services Authority). Where a general practitioner had an age-sex register the two men with dates of birth nearest to that of the case were selected, otherwise the record of the case was located and, starting ten records further on, the first two eligible men selected. Only one control was interviewed, the second being kept as a reserve if the first control could not be interviewed for some reason. In some instances, it was necessary to choose third or fourth controls. If a case could not be interviewed, no attempt was made to interview his matched control.

For both cases and controls, the study was restricted to white men with no previous malignancy or severe mental handicap or psychiatric condition (as determined by consultant or GP). Psychiatric problems arising as a result of diagnosis did not render a case ineligible. Cases diagnosed abroad and controls whose names were still on the GP's list but who had moved out of the study area before control selection were also excluded. The cases and controls were first contacted by letter, from the consultant or GP respectively, and informed of the purpose of the study. They were told that the interview would cover a wide range of topics. The letter was followed by a telephone call within 3 weeks if no reply had been received, or by a visit from interviewing staff.

*Interviewing*

Interviewing took place between June 1984 and April 1988. Each case-control pair was seen by the same female interviewer, and most interviews took place in GP surgeries, although occasionally at the man's home, place of work or in hospital. In 95% of both the case and control interviews no-one else was present apart from interviewer and interviewee. Interviewers attended training sessions together annually.

*Data*

The interview, identical for cases and controls apart from a final section on events leading up to diagnosis in cases, took 60-90 min to complete, and included questions on personal history, sexual development and behaviour, medical history, marital history and children, lifestyle including sport and exercise, occupational history and exposures, and family history (see Appendix 1 for a detailed list of topics covered). Every control was given a 'pseudodiagnosis' date, the date on which he was exactly the same age as his matched case was at diagnosis. A 'reference age' was defined as the age of the case and control 1 year before the diagnosis/pseudodiagnosis date. Most data collected referred to events happening before the reference age.

At the end of the interview, each man who had a mother alive and under 70 years of age was asked for his permission to contact her with a postal questionnaire. The questionnaire, sent with an explanatory letter to the mother, asked about the mother's obstetric history, particularly with respect to the son included in the study, and about his health as a child.

After interview, and with the interviewee's consent, data on medical history, particularly with respect to cryptorchidism and testicular damage, were abstracted from GP notes by the interviewers using a structured form. For cases, details of their testicular tumour were abstracted from their hospital notes, and a copy of their pathology reports obtained. Hospital notes were used only to confirm the diagnosis. All other data sources were used to confirm the history of cryptorchidism, hernia and trauma. Otherwise case and control questionnaires only were used.

*Statistical methods*

The statistical analysis used multivariate conditional logistic regression methods for individually matched case-control studies (Breslow & Day, 1980). Relative risks were estimated by odds ratios (OR) with 95% confidence intervals (CI). A case-control pair was excluded if the information for either the case or the control was not known for the variable in question. Significance tests (*P*-values) quoted are two sided. Tests for trend were calculated either across categories or using recorded levels for continuous variables as appropriate (see footnotes to tables). The only adjustment factors were a history of undescended testis and inguinal hernia at less than 15 years of age. Odds ratios are presented both unadjusted and adjusted for these variables.

### Results

A total of 863 eligible cases were identified and 794 (92.0%) were interviewed. Reasons for non-interview were consultant refusal on grounds of being too ill (12), case refusal (14), death before interview (27) and migration out of the study area (16). Of the 794 first-selected controls, 61 (7.7%) had moved from their registered address, and 609 of the remaining 733 (83.1%) were interviewed. Reasons for non-interview were GP refusal (14), control refusal (84) and failure to trace (26). Of the 185 first-selected controls that could not be interviewed, 142 (76.8%) second-choice controls were successfully interviewed and 43 subsequent choices.

Physical characteristics (height, weight, Quetelet index and 'handedness' for a number of activities) were all unrelated to testicular cancer risk. Height and weight at reference age are shown in Table I, as is the hand used for writing. For Quetelet index at reference age no trend with testicular cancer risk was apparent ($\chi^2_1 = 0.29$, $P = 0.59$). Similar results (not shown) were found for weight and Quetelet index at age 20, and for 'handedness' related to other activities.

There was little evidence of a relationship between cigarette smoking (Table I) and testicular cancer risk. For ever-smoking the OR was 1.18 (95% CI 0.96-1.46), and there was only weak evidence of an increasing trend in relative risks with smoking intensity ($\chi^2_1 = 2.58$, $P = 0.11$) [average number of cigarettes per day multiplied by number of years smoked divided by (reference age minus 13 years)] but none with age at start of regular smoking (data not shown). Alcohol consumption both at reference age and at age 20 (Table I) was unrelated to testicular cancer risk.

Factors related to physical trauma to the testis and to testicular temperature are shown in Table II. Injury to the testis resulting in at least 1 day's absence from work or school was significantly associated with cancer risk (OR = 1.84, 95% CI 1.03-3.27). Seventy-nine injuries reported at interview as resulting in a GP and/or hospital visit were reviewed by us (D.F. and C.E.D.C.) using the men's questionnaires, the mothers' questionnaires and the GP note abstracts. In ten instances (eight cases, two controls) there was evidence that the injury was not to the testis. Twenty-five of the remaining 69 reported injuries were confirmed by the mother and/or the GP, in 17 instances neither GP notes nor mother's questionnaire were available, and in 27 (13 cases, 14 controls), although either one or other course was available, the injury was not mentioned. The odds ratio for confirmed testicular trauma requiring a GP and/or hospital visit based on 25 injuries was 2.05 (95% CI 0.88-4.76). There was substantial variation in severity. Kicks associated with sport were the commonest cause of injury (15 cases, 11 controls), followed by kicks from humans (unassociated with sport) or animals (nine cases, seven controls) and motorbike accidents (seven cases, one control). A number of severe injuries resulted from falling astride fences or walls and one case was impaled on a tractor while sky-diving. Twenty-one cases and three controls had their first injury within 2 years of the diagnosis/pseudodiagnosis date (OR = 7.46, 95% CI 2.20-25.26). The odds ratio for a longer duration of elapsed time since first injury was 2.00 (95% CI 1.54-2.61) with no evidence of any trend ($\chi^2_1 = 0.22$, $P = 0.64$) (Table II).

The testis may also be injured by a torsion (three cases and two controls, OR = 0.96, 95% CI 0.15-6.22). The result of severe damage may be atrophy; four cases and one control reported an atrophic testis in answer to an open-ended question on 'other testicular problems' (OR = 2.74, 95% CI 0.29-25.79). Atrophy may be associated with cryptorchidism, but none of the four cases had a history of cryptochidism, unlike the control. In two of the four cases the tumour arose in the atrophic testis. For atrophy without cryptochidism the difference remained statistically non-significant ($P = 0.13$). Another cause of testicular damage is orchitis, often due to post-pubertal mumps. All forms of orchitis had an odds ratio of 1.93 (95% CI 0.64-5.81). Mumps orchitis was reported by five cases and four controls (OR = 1.27, 95% CI 0.33-4.94) and orchitis not associated with mumps by five cases and one control (OR = 4.35, 95% CI 0.50-37.81).

There was no clear relationship between type (jockey versus boxer shorts) or tightness of underpants and testicular cancer risk, but testicular cancer patients were more likely to have worn nylon underpants at some time than always to have worn cotton (OR = 1.81, 95% CI 1.04-3.14, for always nylon and OR = 1.25, 95% CI 1.01-1.55, for sometimes cotton and sometimes nylon compared with cotton alone). A similar relationship held at age 20 (OR = 1.64, 95% CI 1.13-2.38, and OR = 1.35, 95% CI 1.04-1.73, for always nylon and both fabrics respectively). The wearing of tight jeans or trousers was unrelated to testicular cancer risk. Odds ratios were close to unity for being 'hot and sweaty in the groin area' whether the question related to reference age or age 20 (data not shown). Bath water temperature was unrelated to testicular cancer risk, as was the proportion of life spent living in centrally heated accommodation.

Sporting activities were grouped into broad categories: contact sports (football, rugby, hockey, American football, lacrosse); racquet sports; water sports; cycling and horse-riding; athletics; cricket, baseball and rounders; and martial arts. The playing of contact sports 1 year before diagnosis had an associated OR of 0.73 (95% CI 0.55–0.97). At age 20 the odds ratio for playing contact sports was 0.80 (95% CI 0.64–1.00). Water sports at ages 16 and 20 were also protective (OR = 0.74, 95% CI 0.58–0.96, at age 16 and OR = 0.74; 95% CI 0.56–0.98, at age 20) but less so at reference age (OR = 0.87, 95% CI 0.66–1.16). Athletic activity was also protective but it related only to reference age (OR = 0.70, 95% CI 0.51–0.97). Martial arts appeared to have a strongly protective effect (OR = 0.42, 95% CI 0.21–0.82, at 16; OR = 0.52, 95% CI 0.29–0.94, at 20; OR = 0.67, 95% CI 0.29–1.54, at reference age). Cycling or horse-riding, racquet sports, and cricket, baseball and rounders were unrelated to testicular cancer risk.

The relative risk associated with ever having had a sexually transmitted disease was 2.22 (95% CI 1.46–3.39) (Table III). Genital herpes (OR = 1.60, 95% CI 0.52–4.89), gonorrhoea (OR = 1.93, 95% CI 1.02–3.63) and 'other' sexually transmitted diseases (OR = 2.27, 95% CI 1.33–3.86) and a history of rashes on the external genitalia (OR = 1.18, 95% CI 0.92–1.52) all had raised odds ratios. There was no clear relationship between the age at which these infections were acquired and testicular cancer risk. We looked at the interval between first reported sexually transmitted disease and diagnosis; the odds ratios for within 5 years, 5–9 years and 10 or more years were 4.69 (95% CI 1.90–11.58), 1.08 (95% CI 0.50–2.33) and 2.18 (95% CI 1.19–3.99) respectively.

Of the other medical problems considered, including the common childhood infectious diseases (not shown), severe acne, atopy, mumps (pre and post age 15), glandular fever, a hydrocele or varicocele or hypospadias, none showed any relationship with risk of testicular cancer (Table III).

Neither social class of the respondent nor of his father (when the respondent was 14 years old) was related to risk of testicular cancer (Table IV). Subdividing at the median age (31 years), there was no social class effect in either subgroup. Occupations were subdivided into 16 standard socioeconomic groups (OPCS, 1980). Employment for five or more years in 'literature/art/sport' had an odds ratio of 2.30 (95% CI 0.79–6.65), and there was an apparent protective effect of

**Table I** Personal characteristics and lifestyle

| Variable | Response | Cases No. | Cases (%) | Controls No. | Controls (%) | Odds ratios Unadjusted | Odds ratios Adjusted[a] (95% CI) |
|---|---|---|---|---|---|---|---|
| Height at reference age (cm)[b] | <170 | 77 | (9.7) | 70 | (8.8) | 1.00 | 1.00 |
| | 170–179 | 340 | (42.9) | 376 | (47.4) | 0.83 | 0.84 (0.58–1.21) |
| | 180–189 | 335 | (42.2) | 319 | (40.2) | 0.96 | 0.96 (0.66–1.40) |
| | >189 | 41 | (5.2) | 29 | (3.7) | 1.29 | 1.29 (0.71–2.34) |
| | Not known | 1 | | 0 | | | |
| | | | | | | | $\chi^2_{trend}$ = 0.87 ($P$ = 0.35) |
| Weight at reference age (kg)[b] | <60 | 46 | (5.8) | 54 | (6.8) | 1.00 | 1.00 |
| | 60–69 | 267 | (33.8) | 256 | (32.3) | 1.20 | 1.25 (0.80–1.96) |
| | 70–79 | 251 | (31.8) | 274 | (34.6) | 1.06 | 1.07 (0.67–1.69) |
| | 80–89 | 155 | (19.6) | 134 | (16.9) | 1.32 | 1.40 (0.86–2.27) |
| | >89 | 71 | (9.0) | 75 | (9.5) | 1.08 | 1.13 (0.66–1.94) |
| | Not known | 4 | | 1 | | | |
| | | | | | | | $\chi^2_{trend}$ = 0.002 ($P$ = 0.97) |
| Hand used for writing | Right | 688 | (87.2) | 684 | (86.3) | 1.00 | 1.00 |
| | Left | 98 | (12.4) | 106 | (13.4) | 0.93 | 0.95 (0.70–1.28) |
| | Both | 3 | (0.4) | 3 | (0.4) | 0.99 | 1.19 (0.23–6.13) |
| | Not known | 5 | | 1 | | | $\chi^2_2$ = 0.16 ($P$ = 0.92) |
| Ever smoked cigarettes | No | 298 | (37.5) | 328 | (41.3) | 1.00 | 1.00 |
| | Yes | 496 | (62.5) | 466 | (58.7) | 1.18 | 1.18 (0.96–1.46) |
| | | | | | | | $\chi^2_1$ = 2.42 ($P$ = 0.12) |
| Smoking intensity[b] | None | 298 | (37.5) | 328 | (41.4) | 1.00 | 1.00 |
| | <10 | 194 | (24.4) | 198 | (25.0) | 1.07 | 1.09 (0.84–1.41) |
| | 10–19 | 222 | (28.0) | 196 | (24.7) | 1.27 | 1.28 (0.98–1.66) |
| | 20–29 | 54 | (6.8) | 43 | (5.4) | 1.40 | 1.34 (0.86–2.09) |
| | ≥30 | 26 | (3.3) | 28 | (3.5) | 1.04 | 1.00 (0.56–1.76) |
| | Not known | 0 | | 1 | | | |
| | | | | | | | $\chi^2_{trend}$ = 2.58 ($P$ = 0.11) |
| Alcohol consumed per week at reference age (g)[b,c] | None | 92 | (11.6) | 101 | (12.7) | 1.00 | 1.00 |
| | <68.8 | 150 | (18.9) | 131 | (16.5) | 1.25 | 1.26 (0.86–1.83) |
| | 68.8– | 147 | (18.5) | 131 | (16.5) | 1.22 | 1.23 (0.85–1.79) |
| | 124.6– | 130 | (16.4) | 157 | (19.8) | 0.89 | 0.87 (0.60–1.28) |
| | 211.2– | 135 | (17.0) | 135 | (17.0) | 1.09 | 1.06 (0.72–1.56) |
| | ≥364.7 | 140 | (17.6) | 139 | (17.5) | 1.11 | 1.13 (0.77–1.66) |
| | | | | | | | $\chi^2_{trend}$ = 0.68 ($P$ = 0.41) |
| Alcholol consumed per week at age 20 (g)[b,c] | None | 63 | (8.5) | 69 | (9.4) | 1.00 | 1.00 |
| | <88.0 | 153 | (20.8) | 124 | (16.8) | 1.34 | 1.38 (0.90–2.12) |
| | 88.0– | 128 | (17.4) | 153 | (20.8) | 0.91 | 0.96 (0.62–1.48) |
| | 176.0– | 122 | (16.6) | 120 | (16.3) | 1.10 | 1.20 (0.76–1.90) |
| | 316.8– | 139 | (18.9) | 135 | (18.3) | 1.11 | 1.10 (0.71–1.72) |
| | ≥508.5 | 132 | (17.9) | 136 | (18.5) | 1.04 | 1.08 (0.69–1.69) |
| | Reference age <20 | 57 | | 57 | | | |
| | | | | | | | $\chi^2_{trend}$ = 0.13 ($P$ = 0.72) |

[a]Adjusted for cryptorchidism, and inguinal hernia at under 15 years of age. See text for definition of smoking intensity. [b]Tests for trend on actual values. [c]Consumption divided into quintiles. Percentages given are for those with known response.

working in 'materials/processing excluding metals and electrical goods' (OR = 0.60, 95% CI 0.41–0.87). Testicular cancer risk was slightly higher in those resident at some time in rural areas compared with those resident always in urban areas (OR = 1.26, 95% CI 0.96–1.65).

### Discussion

The results reported here are from the largest interview-based case–control study of the aetiology of testicular cancer ever carried out. The study was population based and the response rate from cases high (92.0%). The method of control selection from general practitioners' lists could lead to some dissimilarities between cases and controls: cases would be registered by virtue of their illness even had they not been registered previously, whereas young, unmarried, healthy men or those moving frequently would be selectively less likely to be registered than the married, unhealthy and static. With regard to most of the factors reported in this paper, it is hard to see how this selection effect would lead to bias. The response rate among controls was 83.1%, only slightly

**Table II** Injurites to testes and testicular temperature

| Variable | Response | Cases No. | Cases (%) | Controls No. | Controls (%) | Odds ratios Unadjusted | Odds ratios Adjusted[a] (95% CI) |
|---|---|---|---|---|---|---|---|
| Time between testicular injury and reference age[b,c] | No injury | 503 | (65.0) | 606 | (78.7) | 1.00 | 1.00 |
| | ≤1 year | 21 | (2.7) | 3 | (0.4) | 7.47 | 7.46 (2.20–25.26) |
| | >1 year | 250 | (32.3) | 161 | (20.9) | 2.03 | 2.00 (1.54–2.61) |
| | Not known | 20 | | 24 | | | |
| | | | | | | | $\chi^2_{trend}$ = 0.22 ($P$ = 0.64) |
| Testis or groin injury resulting in at least one day off work or school[b] | No | 759 | (95.6) | 775 | (97.6) | 1.00 | 1.00 |
| | Yes | 35 | (4.4) | 19 | (2.4) | 1.89 | 1.84 (1.03–3.27) |
| Testis or groin injury for which consulted GP[b] | No | 765 | (96.3) | 770 | (97.0) | 1.00 | 1.00 |
| | Yes | 29 | (3.7) | 24 | (3.0) | 1.22 | 1.20 (0.68–2.10) |
| Testis or groin injury for which went to hospital[b] | No | 769 | (96.9) | 779 | (98.1) | 1.00 | 1.00 |
| | Yes | 25 | (3.1) | 15 | (1.9) | 1.77 | 1.68 (0.85–3.33) |
| Testicular torsion | No | 791 | (99.6) | 792 | (99.7) | 1.00 | 1.00 |
| | Yes | 3 | (0.4) | 2 | (0.3) | 1.50 | 0.96 (0.15–6.22) |
| Testicular atrophy | No | 790 | (99.5) | 793 | (99.9) | 1.00 | 1.00 |
| | Yes | 4 | (0.5) | 1 | (0.1) | 4.00 | 2.74 (0.29–25.79) |
| Orchitis | No | 784 | (98.7) | 789 | (99.4) | 1.00 | 1.00 |
| | Yes | 10 | (1.3) | 5 | (0.6) | 2.00 | 1.93 (0.64–5.81) |
| Underpants worn up to reference age | Jockey,Y-fronts | 692 | (88.0) | 665 | (84.6) | 1.00 | 1.00 |
| | Boxer | 19 | (2.4) | 17 | (2.2) | 1.04 | 1.02 (0.52–2.01) |
| | Either | 75 | (9.5) | 104 | (13.2) | 0.68 | 0.70 (0.50–0.97) |
| | Not known | 8 | | 8 | | | |
| | | | | | | | $\chi^2_2$ = 4.74 ($P$ = 0.094) |
| Tight fitting underpants worn up to reference age | Never | 505 | (64.3) | 533 | (67.8) | 1.00 | 1.00 |
| | Some ages | 137 | (17.5) | 118 | (15.0) | 1.22 | 1.22 (0.92–1.63) |
| | Always | 143 | (18.2) | 135 | (17.2) | 1.12 | 1.06 (0.81–1.38) |
| | Not known | 9 | | 8 | | | |
| | | | | | | | $\chi^2_{trend}$ = 0.55 ($P$ = 0.46) |
| Material of underpants worn up to reference age | Cotton | 352 | (45.5) | 399 | (51.2) | 1.00 | 1.00 |
| | Nylon | 41 | (5.3) | 29 | (3.7) | 1.86 | 1.81 (1.04–3.14) |
| | Either | 380 | (49.2) | 352 | (45.1) | 1.23 | 1.25 (1.01–1.55) |
| | Not known | 21 | | 14 | | | |
| | | | | | | | $\chi^2_2$ = 7.64 ($P$ = 0.022) |
| Tight trousers or jeans worn up to reference age | Never | 301 | (38.1) | 299 | (37.8) | 1.00 | 1.00 |
| | Some ages | 259 | (32.8) | 263 | (33.2) | 0.98 | 0.97 (0.77–1.23) |
| | Always | 230 | (29.1) | 229 | (29.0) | 1.00 | 0.96 (0.74–1.25) |
| | Not known | 4 | | 3 | | | |
| | | | | | | | $\chi^2_{trend}$ = 0.10 ($P$ = 0.76) |
| Temperature of bath water up to reference age | Always tepid | 95 | (12.1) | 67 | (8.5) | 1.00 | 1.00 |
| | Always hot | 353 | (44.9) | 382 | (48.3) | 0.66 | 0.69 (0.48–0.98) |
| | Always very hot | 145 | (18.4) | 143 | (18.1) | 0.72 | 0.77 (0.52–1.15) |
| | No baths taken | 17 | (2.2) | 18 | (2.3) | 0.65 | 0.63 (0.28–1.42) |
| | Mixture of temperatures | 177 | (22.5) | 181 | (22.9) | 0.69 | 0.76 (0.52–1.12) |
| | Not known | 7 | | 3 | | | |
| | | | | | | | $\chi^2_4$ = 4.80 ($P$ = 0.31) |
| Percentage of life to reference age in centrally heated accommodation[c] | None | 154 | (20.9) | 182 | (23.7) | 1.00 | 1.00 |
| | <25 | 201 | (27.2) | 183 | (23.8) | 1.34 | 1.41 (1.03–1.94) |
| | 25–49 | 217 | (29.4) | 208 | (27.0) | 1.28 | 1.30 (0.95–1.78) |
| | 50–74 | 87 | (11.8) | 94 | (12.2) | 1.08 | 1.02 (0.70–1.48) |
| | 75–99 | 30 | (4.1) | 42 | (5.5) | 0.77 | 0.79 (0.46–1.38) |
| | 100 | 49 | (6.6) | 60 | (7.8) | 0.83 | 0.81 (0.50–1.33) |
| | Not known | 56 | | 25 | | | |
| | | | | | | | $\chi^2_{trend}$ = 0.82 ($P$ = 0.37) |

[a]Adjusted for cryptorchidism, and inguinal hernia at under 15 years of age. Percentages given are for those with known response. [b]Assessment using GP notes and mothers' questionnaire as well as questionnaire data. [c]Tests for trend on actual values.

lower than for a case–control study of breast cancer in young women using a similar methodology (UK National Case–Control Study Group, 1989). Other potential sources of bias are discussed elsewhere (UK Testicular Cancer Study Group, 1994).

We found little effect of personal characteristics or lifestyle on testicular cancer risk. Two other studies have considered smoking (Henderson *et al.*, 1979; Coldman *et al.*, 1982), and neither found any evidence of any effect; the evidence from our data is weak. Likewise, handedness is unrelated to risk in our data. Swerdlow *et al.* (1987) found a lower proportion of cases than controls to be left-handed or ambidextrous and suggested a hypothesis relating handedness to testicular cancer risk mediated by maternal hormone levels *in utero*.

There has been considerable debate as to the relevance of testicular trauma to tumour development (Field, 1963). Our analysis of the time between injury and diagnosis/pseudo-diagnosis demonstrated that substantially more of the injuries reported within 2 years of diagnosis occurred in cases than controls (21 *vs* 3; OR = 7.46). Although trauma could have a late-stage effect due to increased trauma-related mitotic activity in an already damaged cell, alternative explanations are either that cases selectively recall more injuries than controls in the period immediately prior to diagnosis, or that the injury led directly to the discovery of the tumour, or that the injury may have accelerated the growth of the tumour. There was certainly some evidence of recall bias in that when checking the trauma reported at interview with data from the mothers' and GPs' notes, proportionally more of the reports from the cases than from the controls were not confirmed, and proportionately more of the cases than control men reported testicular injuries that, on checking the other sources, were not actually to the testis. For longer periods of time since first trauma odds ratios were all statistically significant and consistently about 2-fold. Our results for trauma are consistent with results reported in case–control studies by Coldman *et al.* (1982), Morris Brown *et al.* (1987) and Haughey *et al.* (1989). Swerdlow *et al.* (1987) had an injury reported spontaneously by six cases and one control. Orchidopexy may also be a cause of physical trauma to the testis.

There are other causes of damage to the testis, particularly orchitis and testicular torsion. Testicular torsion as a cause of testicular cancer was suggested by Chilvers *et al.* (1987) in their analysis of 10 years' data from the Royal Marsden Hospital, but our results do not support an association. Orchitis is rare, and other studies have reported raised relative risks (Mills *et al.*, 1984; Morris Brown *et al.*, 1987; Swerdlow *et al.*, 1987) but based on very small numbers. Post-pubertal mumps is a common cause of orchitis, but we found no relationship with mumps. At its most severe, trauma to the testis may result in testicular atrophy. Two case–control studies included direct questions on testicular atrophy, and both reported an association (Swerdlow *et al.*, 1987; Haughey *et al.*, 1989). There is thus some evidence of a relationship between atrophy and the development of a germ cell tumour. Sixteen per cent of cases in Haughey *et al.*'s US study and 3.5% in Swerdlow *et al.*'s UK study reported testicular atrophy compared with only 0.5% in ours. The small proportion in our study is likely to be because we did not ask a specific question about atrophy. A possible explanation of the discrepancy between Haughey *et al.*'s and Swerdlow *et al.*'s results could be that, in the US, cases might be more likely to be told of the relationship during their hospital treatment.

Sports injuries may also be relevant to testicular trauma. The strongest prior hypothesis relates to cycling and horse-riding where the possibility of trauma to the testis is readily apparent. Both Coldman *et al.* (1982) and Haughey *et al.* (1989) found raised relative risks associated with these activities, but we found no evidence of any such effect. Haughey *et al.* (1989) found a slightly raised relative risk associated with contact sports, as did Coldman *et al.* (1982) for soccer. We found a protective effect associated with

**Table III** Sexually transmitted diseases, and history of other illness

| Variable | Response | Cases No. | Cases (%) | Controls No. | Controls (%) | Odds ratios Unadjusted | Odds ratios Adjusted[a] (95% CI) |
|---|---|---|---|---|---|---|---|
| Ever had any sexually transmitted disease | No | 715 | (90.4) | 755 | (95.1) | 1.00 | 1.00 |
| | Yes | 76 | (9.6) | 39 | (4.9) | 2.15 | 2.22 (1.46–3.39) |
| | Not known | 3 | | 0 | | | |
| Ever had genital herpes | No | 784 | (99.0) | 789 | (99.4) | 1.00 | 1.00 |
| | Yes | 8 | (1.0) | 5 | (0.6) | 1.60 | 1.60 (0.52–4.89) |
| | Not known | 2 | | 0 | | | |
| Ever had gonorrhoea | No | 761 | (96.2) | 778 | (98.0) | 1.00 | 1.00 |
| | Yes | 30 | (3.8) | 16 | (2.0) | 1.93 | 1.93 (1.02–3.63) |
| | Not known | 3 | | 0 | | | |
| Ever had any other sexually transmitted disease | No | 744 | (94.1) | 770 | (97.0) | 1.00 | 1.00 |
| | Yes | 47 | (5.9) | 24 | (3.0) | 2.14 | 2.27 (1.33–3.86) |
| | Not known | 3 | | 0 | | | |
| Ever had acne (requiring medical treatment) | No | 719 | (90.6) | 721 | (90.8) | 1.00 | 1.00 |
| | Yes | 75 | (9.4) | 73 | (9.2) | 1.03 | 1.07 (0.75–1.52) |
| Ever had atopy (hay fever, asthma or eczema) | No | 566 | (71.3) | 577 | (72.7) | 1.00 | 1.00 |
| | Yes | 228 | (28.7) | 217 | (27.3) | 1.07 | 1.09 (0.87–1.37) |
| Ever had mumps and age at first attack | No | 296 | (42.5) | 312 | (44.9) | 1.00 | 1.00 |
| | <15 years | 385 | (55.2) | 360 | (51.8) | 1.17 | 1.17 (0.92–1.48) |
| | ⩾15 years | 16 | (2.3) | 23 | (3.3) | 0.86 | 0.77 (0.37–1.60) |
| | Not known | 97 | | 99 | | | |
| Glandular fever | No | 727 | (92.5) | 716 | (91.0) | 1.00 | 1.00 |
| | Yes | 59 | (7.5) | 71 | (9.0) | 0.82 | 0.84 (0.59–1.22) |
| | Not known | 8 | | 7 | | | |
| Hydrocele varicocele | No | 776 | (97.7) | 780 | (98.2) | 1.00 | 1.00 |
| | Yes | 18 | (2.3) | 14 | (1.8) | 1.29 | 1.09 (0.53–2.24) |
| Hypospadias | No | 793 | (99.9) | 793 | (99.9) | 1.00 | 1.00 |
| | Yes | 1 | (0.1) | 1 | (0.1) | 1.00 | 0.54 (0.03–9.98) |

[a]Adjusted for cryptorchidism, and inguinal hernia at under 15 years of age. Percentages given are for those with known response.

contact sports and some other sporting activities. Any effect of sport where the testis is not likely to be directly traumatised (i.e. not riding or cycling), could be the antithesis of the sedentary lifestyle associated with an increased risk which we report elsewhere (UK Testicular Cancer Study Group, 1994). We have also reported an inverse association between testicular cancer risk and total hours of exercise each week at reference age and at age 20 (UK Testicular Cancer Study Group, 1994). The reported protective effects of sports remained, however, after adjustment for total exercise.

A relationship between sexually transmitted diseases and testicular germ cell tumours has not been reported previously (Coldman *et al.*, 1982; Moss *et al.*, 1986; Morris Brown *et al.*, 1987; Swerdlow *et al.*, 1987). We report raised relative risks for all types of sexually transmitted disease, although only the odds ratios for gonorrhoea and 'other' sexually transmitted diseases were statistically significant. The sensitive nature of such questions makes them particularly susceptible to reporting bias, and there was some evidence of this in that the highest odds ratio was for a sexually transmitted disease within the previous 5 years. The odds ratio for a sexually transmitted disease diagnosed 10 or more year ago was, however, also statistically significantly increased. We included questions on a wide range of other medical conditions which had been previously studied but found no significant associations. Of the three previous studies including questions on atopy, two found no association (Henderson *et al.*, 1979; Morris Brown *et al.*, 1987) and the third (Swerdlow *et al.*, 1987) found a significantly increased relative risk (OR = 1.8, 95% CI 1.1–3.1). Two studies have suggested a protective effect of 'treated' acne (Depue *et al.*, 1983; Morris Brown *et al.*, 1987), while two others found no effect of acne (Henderson *et al.*, 1979; Moss *et al.*, 1986). Most other authors report, as we do, a lack of association with childhood infectious diseases (Henderson *et al.*, 1979; Coldman *et al.*, 1982; Moss *et al.*, 1986; Swerdlow *et al.*, 1987; Morris Brown *et al.*, 1987; Haughey *et al.*, 1989), but Loughlin *et al.* (1980) in their pilot case–control study reported a relative risk for mumps of 5.9 ($P = 0.07$) from a questionnaire to mothers of cases and controls. Mills *et al.* (1984) suggested that residence in a rural community might lead to lack of early immunity from infections and an increased risk as a result of some viral infection acquired at an unusually late age. We found a non-significant increased risk of testis cancer in those resident at some time in rural areas. Some authors have also reported this (Lipworth & Dayan, 1969; Talerman *et al.*, 1974; Graham *et al.*, 1977), but not others (Coldman *et al.*, 1982; Waterhouse *et al.*, 1982; Morris Brown & Pottern, 1984; Moss *et al.*, 1986). A more specific hypothesis relating to Epstein–Barr virus has been proposed by Newell *et al.* (1984). A question on glandular fever (infectious mononucleosis) has been included in two case–control studies; Swerdlow *et al.* (1987) found, as we do, no evidence of a relationship with testicular cancer risk, but Moss *et al.* (1986) found a protective effect for seminoma only (OR = 0.3, $P = 0.009$).

**Table IV** Social class, employment history and residence

| Variable | Response | *Cases* No. | (%) | *Controls* No | (%) | *Odds ratios* Unadjusted | Adjusted[a] (95% CI) |
|---|---|---|---|---|---|---|---|
| Social class | I | 46 | (5.8) | 60 | (7.6) | 1.00 | 1.00 |
| | II | 212 | (26.7) | 202 | (25.4) | 1.35 | 1.29 (0.83–2.00) |
| | III NM | 112 | (14.1) | 95 | (12.0) | 1.49 | 1.38 (0.86–2.22) |
| | III M | 264 | (33.2) | 280 | (35.3) | 1.21 | 1.17 (0.76–1.81) |
| | IV | 93 | (11.7) | 87 | (11.0) | 1.37 | 1.36 (0.81–2.26) |
| | V | 28 | (3.5) | 30 | (3.8) | 1.20 | 1.14 (0.58–2.26) |
| | Miscellaneous[b] | 39 | (4.9) | 40 | (5.0) | 1.21 | 1.38 (0.64–2.97) |
| | | | | | | | $\chi^2_{trend} = 0.01$ ($P = 0.91$) |
| Social class of father | I | 35 | (4.4) | 37 | (4.7) | 1.00 | 1.00 |
| | II | 191 | (24.1) | 197 | (24.8) | 1.04 | 1.12 (0.68–1.87) |
| | III NM | 66 | (8.3) | 66 | (8.3) | 1.05 | 1.16 (0.65–2.05) |
| | III M | 347 | (43.7) | 316 | (39.8) | 1.18 | 1.28 (0.77–2.12) |
| | IV | 90 | (11.3) | 82 | (10.3) | 1.16 | 1.27 (0.73–2.23) |
| | V | 37 | (4.7) | 43 | (5.4) | 0.92 | 1.07 (0.54–2.09) |
| | Miscellaneous[b] | 28 | (3.5) | 53 | (6.7) | 0.56 | 0.62 (0.32–1.20) |
| | | | | | | | $\chi^2_{trend} = 0.85$ ($P = 0.36$) |
| *Employed 5 or more years in* | | | | | | | |
| Professional management | | 53 | (6.7) | 56 | (7.1) | 0.93 | 0.97 (0.63–1.49) |
| Professional – education/welfare/health | | 29 | (3.7) | 32 | (4.0) | 0.90 | 0.93 (0.54–1.59) |
| Literature/art/sport | | 14 | (1.8) | 6 | (0.8) | 2.60 | 2.30 (0.79–6.65) |
| Professional – science/engineering/technology | | 59 | (7.4) | 62 | (7.8) | 0.94 | 0.94 (0.64–1.39) |
| Managerial | | 60 | (7.6) | 55 | (6.9) | 1.11 | 1.08 (0.71–1.63) |
| Clerical | | 55 | (6.9) | 44 | (5.5) | 1.28 | 1.26 (0.82–1.93) |
| Sales | | 43 | (5.4) | 32 | (4.0) | 1.41 | 1.33 (0.81–2.21) |
| Security | | 14 | (1.8) | 15 | (1.9) | 0.93 | 0.79 (0.37–1.68) |
| Personal services | | 20 | (2.5) | 20 | (2.5) | 1.00 | 1.06 (0.55–2.06) |
| Farming/fishing | | 16 | (2.0) | 11 | (1.4) | 1.46 | 1.48 (0.67–3.24) |
| Materials processing (excluding metal and electrical) | | 55 | (6.9) | 85 | (10.7) | 0.61 | 0.60 (0.41–0.87) |
| Processing/metal/electrical | | 141 | (17.8) | 140 | (17.6) | 1.01 | 1.04 (0.78–1.37) |
| Painting/repetitive assembly/product inspection/packaging | | 33 | (4.2) | 32 | (4.0) | 1.03 | 1.03 (0.63–1.69) |
| Construction/mining | | 46 | (5.8) | 32 | (4.0) | 1.52 | 1.47 (0.90–2.41) |
| Transport operating | | 53 | (6.7) | 59 | (7.4) | 0.89 | 0.88 (0.59–1.32) |
| Miscellaneous | | 64 | (8.1) | 79 | (9.9) | 0.78 | 0.79 (0.55–1.14) |
| Area of residence | | | | | | | |
| Always urban | | 467 | (58.8) | 491 | (61.8) | 1.00 | 1.00 |
| Ever rural | | 327 | (41.2) | 303 | (38.2) | 1.25 | 1.26 (0.96–1.65) |
| Rural <5 years | | 85 | (10.7) | 68 | (8.6) | 1.36 | 1.41 (0.98–2.04) |
| 5–15 years | | 94 | (11.8) | 92 | (11.6) | 1.14 | 1.14 (0.79–1.63) |
| >15 years | | 148 | (18.6) | 143 | (18.0) | 1.21 | 1.19 (0.80–1.78) |

[a]Adjusted for cryptorchidism, and inguinal hernia at under 15 years of age. Percentages given are for those with known response. [b]Miscellaneous category excluded from trend test.

There has been much debate about the role of testicular temperature in the aetiology of testicular cancer, arising primarily as a possible explanation for the well-established relationship with cryptorchidism. We found no evidence of an effect of testicular temperature on cancer risk. Loughlin *et al.* (1980) first suggested that the wearing of tight-fitting underpants might be associated with an increased risk (OR = 3.1). In subsequent studies the evidence was unconvincing (Moss *et al.*, 1986; Morris Brown *et al.*, 1987; Haughey *et al.*, 1989; Karagas *et al.*, 1989), although Karagas *et al.* (1989) did find slight evidence of an increasing trend in relative risk with number of months each year wearing long underwear. Haughey *et al.* (1989) found an increased risk associated with exposure to heat at work (OR = 1.74, 95% CI 1.2–2.6), but conversely Karagas *et al.* (1989) found that the wearing of heat-resistant clothing at work (implying heat exposure) had an odds ratio close to unity (OR = 0.9, 95% CI 0.3–2.8). Bathing (as distinct from showering) had an odds ratio of 3.1 (95% CI 1.5–9.9) in Haughey *et al.*'s case–control study, but 0.9 (95% CI 0.5–1.6) according to Morris Brown *et al.* (1987). Use of saunas and 'hot-tubs' has shown no effect (Haughey *et al.*, 1989; Karagas *et al.*, 1989).

Testicular temperature may also be related to the amount of time spent seated each day and hence to the relationship of social class to testicular cancer risk. A strong relationship between social class and testicular cancer risk (the higher the social class, the greater the risk) has been demonstrated using routinely collected statistics (OPCS, 1981–6). We found no evidence of any social class effect either in the cases and controls themselves or in their fathers. Our method of control selection (using the same general practitioner for the case and control in each pair) would tend to match on social class, but a comparison of the social class of cases and their matched controls suggested that the diluting effect would be small. Moreover, the lack of effect in the fathers suggests that social class is not an important risk factor. Moss *et al.* (1986) found their cases to be of slightly higher social class and better educated than their controls, in spite of some possible overmatching, and Depue *et al.* (1983) found their cases to have a higher social class distribution than that of Los Angeles residents as a whole. Hayes *et al.* (1990) found an increased risk of seminoma for professional occupations (OR = 2.8 95% CI 1.4–5.4). Coldman *et al.* (1982) and Haughey *et al.* (1989) found the social class distribution of cases and controls to be similar. Educational levels in the studies of Morris Brown *et al.* (1987) and Karagas *et al.* (1989) were similar, although Haughey *et al.* (1989) found their cases to be less well educated than their controls. The explanation for the lack of effect in more recent studies may be related to the reduction in lifestyle differences between different socioeconomic groups. Our study, however, found no social class effect in either those younger or those older than the median age, whereas an effect restricted to the older subjects might have been expected.

Individual occupations have been extensively studied, and the possibility of statistically significant results arising by chance when large numbers of tests are carried out has been pointed out by Forman (1989), who summarised the variety of occupations that have been associated with an increased risk. The relative risk for farming (OR = 6.27, 95% CI 1.83–2.15, Mills *et al.*, 1984; OR = 0.4, 95% CI 0.2–0.9, seminoma only, Hayes *et al.*, 1990; OR = 1.48, 95% CI 0.67–3.24, this paper) and for related exposures (fertilisers OR = 2.27, 95% CI 1.30–5.0, Haughey *et al.*, 1989; pesticides OR = 1.2, Hayes *et al.*, 1990) show little consistency. A similar lack of consistency was found for filling station service (Coldman *et al.*, 1982; Hayes *et al.*, 1990). Any relationship between social class and testicular cancer risk as found by some authors is much more likely to be related to lifestyle than to occupation *per se* (Forman, 1989).

In this, the largest aetiological study of testicular cancer so far carried out, a wide variety of social, behavioural and medical factors were included in the interview. The strongest findings reported here were 2-fold risks associated with past testicular trauma and with having had a sexually transmitted disease. Sporting activity had a protective effect. This study provides little evidence of an assocation with cigarette smoking and no evidence to support any effects of handedness, autoimmunity, atopy or the wearing of tight underpants or trousers. There were no clear occupational associations nor any association with social class. We find no evidence that testicular temperature is associated with testicular cancer risk.

This study was funded by the Imperial Cancer Research Fund, the Cancer Research Campaign and the Medical Research Council. We thank M. Slattery (Wessex Regional Health Authority), R.G. Skeet (Thames Cancer Registry), F. Landells, L. Jenkinson (ICRF Genetic Epidemiology Lab, Leeds) and F. Ledbetter (Welsh Office) for help in case finding, the family practitioner committees (now FHSAs) who helped with control selection, all the consultants and general practitioners who allowed us to interview their patients and, most of all, the patients and control men who so willingly helped us with the study. The manuscript was prepared by Melanie Cumpston.

**References**

BRESLOW, N.E. & DAY, N.E. (1980). *Statistical Methods in Cancer Research*, Vol. 1, The Analysis of Case–Control Studies. IARC: Lyon.

CHILVERS, C., PIKE, M.C. & PECKHAM, M.J. (1987). Torsion of the testis: a new risk factor for testicular cancer. *Br. J. Cancer*, **55**, 106–107.

CHILVERS, C.E.D. & PIKE, M.C. (1992). Cancer Risk in the Undescended Testicle. *European Urology Update Series*, **1**, 10.

COLDMAN, A.J., ELWOOD, J.M. & GALLAGHER, R.P. (1982). Sports activites and risk of testicular cancer. *Br. J. Cancer*, **46**, 749–756.

DEPUE, R.H., PIKE, M.C. & HENDERSON, B.E. (1983). Estrogen exposure during gestation and risk of testicular cancer. *J Natl Cancer Inst.*, **71**, 1151–1155.

FIELD, T.E. (1963). The role of trauma in the aetiology of testicular neoplasms. *J. R. Army Med. Corps.*, **109**, 58–61.

FORMAN, D. (1989). Epidemiology of testis cancer. In *Urological and Genital Cancer*, Oliver, R.T.D., Blandy, J.P. & Hope-Stone, H.F. (eds) pp. 289–305. Blackwell Scientific Publications: Oxford.

GRAHAM, S., GIBSON, R., WEST, D., SWANSON, M., BURNETT, W. & DAYAL, H. (1977). Epidemiology of cancer of the testis in upstate New York. *J. Natl Cancer Inst.*, **58**, 1225–1261.

HAUGHEY, B.P., GRAHAM, S., BRASURE, J., ZIELEZNY, M., SUFRIN, G. & BURNETT, W.S. (1989). The epidemiology of testicular cancer in upstate New York. *Am. J. Epidemiol.*, **130**, 25–36.

HAYES, R.B., MORRIS BROWN, L. & POTTERN, L.M. (1990). Occupation and risk for testicular cancer: a case control study. *Int. J. Epidemiol.*, **19**, 825–831.

HENDERSON, B.E., BENTON, B., JIN, J., YU, M.C. & PIKE, M.C. (1979). Risk factors for cancer of the testis in young men. *Int. J. Cancer*, **23**, 598–602.

KARAGAS, M.R., WEISS, N.E.,STRADER, C.H. & DALING, J.R. (1989). Elevated intrascrotal temperature and the incidence of testicular cancer in noncryptorchid men. *Am. J. Epid.*, **129**, 1104–1109.

LIPWORTH, L. & DAYAN, A.D. (1969). Rural preponderance of seminoma of the testis. *Cancer*, **23**, 1119–1121.

LOUGHLIN, J.E., ROBBOY, S.J. & MORRISON, A.S. (1980). Risk factors for cancer of the testis. *N. Engl. J. Med.*, **303**, 112–113.

MILLS, P.K., NEWELL, G.R. & JOHNSON, D.E. (1984). Testicular cancer associated with employment in agriculture and oil and natural gas extraction. *Lancet*, **i**, 207–210.

MORRIS BROWN, L.M. & POTTERN, L.M. (1984). Testicular cancer and farming. *Lancet*, **i**, 1356.

MORRIS BROWN, L.M., POTTERN, L.M. & HOOVER, R.N. (1987). Testicular cancer in young men: the search for causes of the epidemic increase in the United States. *J. Epidemiol. Community Hlth.*, **41**, 349–354.

MOSS, A.R., OSMOND, D., BACCHETTI, P., TORTI, M. & GURGIN, V. (1986). Hormonal risk factors in testicular cancer. A case–control study. *Am. J. Epidemiol.*, **124**, 39–51.

NEWELL, G.R., MILLS, P.K. & JOHNSON, D. (1984). Epidemiological comparison of cancer of the testis and Hodgkin's disease among young males. *Cancer*, **54**, 1117–1123.

OFFICE OF POPULATION CENSUSES AND SURVEYS (1981-6). *Cancer Registries of England and Wales. MBI 1978-1982.* HMSO: London.

SCHOTTENFELD, D., WARSHAUER, M.E., SHERLOCK, S., ZAUBER, A.G., LEDER, M. & PAYNE, R. (1980). The epidemiology of testicular cancer in young adults. *Am. J. Epidemiol.*, **112**, 232-246.

SWERDLOW, A.J., HUTTLEY, S.R.A. & SMITH, P.G. (1987). Prenatal and familial associations of testicular cancer. *Br. J. Cancer*, **55**, 571-577.

TALERMAN, A., KAALEN, J.G. & FOKKENS, W. (1974). Rural preponderance of testicular neoplasms. *Br. J. Cancer*, **29**, 176-178.

UK NATIONAL CASE-CONTROL STUDY GROUP (1989). Oral contraceptive use and breast cancer risk in young women. *Lancet*, **i**, 973-982.

UK TESTICULAR CANCER STUDY GROUP (1994). The aetiology of testicular cancer: association with congenital abnormalities, age at puberty, infertility and exercise. *Br. Med. J.*, **308**, 1393-1399.

WATERHOUSE, J., MUIR, C., SHANMUGARETNAM, K. & POWELL, J. (1982). (eds). *Cancer Incidence in Five Continents*, Vol. 4. IARC: Lyon.

### Appendix 1: The questionnaire

Case and control unless otherwise stated

A. Personal history

- Residence history

- Martial status

- Height and weight

- Development of secondary sexual characteristics

B. Medical history

- History of childhood infections and other conditions

- Circumcision

- Hernia

- Testicular trauma

- Undescended testis

- History of selected adult diseases

- Dyslexia

- Handedness

C. Reproductive history

- Difficulty in conception, fertility

- Vasectomy

D. Smoking

E. Alcohol

F. Medication and irradiation

- Hormone treatment

- Long-term medication

- X-ray exposure

G. Occupational history
- All jobs for more than 3 months
- Own and father's social class

H. Chemical and other exposures
- Exposure to various substances occupationally or at home

I. Transport

- Bicycle/motorbike/horse etc

J. Clothing and testicular temperature

- Underpants

- Tight jeans

- Bathwater temperature

- Central heating

K. Sexual history

- Masturbation

- Age at first intercourse

- Heterosexual and homosexual partners

- Sexually transmitted diseases

L. Sport and exercise

- Time spent sitting

- Vigorous exercise: age 16, 20 reference age

- Active sports: age 16, 20, reference age

M. Family history

- Cancer

- Testicular problems

- Undescended testis

N. (Cases only)
- Events leading to diagnosis of testis cancer